# A Transient Printed Soil Decomposition Sensor Based on a Biopolymer Composite Conductor

**DOI:** 10.1002/advs.202205785

**Published:** 2022-12-11

**Authors:** Madhur Atreya, Stacie Desousa, John‐Baptist Kauzya, Evan Williams, Austin Hayes, Karan Dikshit, Jenna Nielson, Abigail Palmgren, Sara Khorchidian, Shangshi Liu, Anupam Gopalakrishnan, Eloise Bihar, Carson J. Bruns, Richard Bardgett, John N. Quinton, Jessica Davies, Jason C. Neff, Gregory L. Whiting

**Affiliations:** ^1^ Paul M. Rady Department of Mechanical Engineering University of Colorado Boulder 1111 Engineering Drive, UCB 427 Boulder CO 80309‐0427 USA; ^2^ Environmental Studies University of Colorado Boulder 4001 Discovery Drive, 397 UCB Boulder CO 80303‐0397 USA; ^3^ Department of Electrical Computer and Energy Engineering University of Colorado Boulder 1111 Engineering Dr, 425 UCB Boulder CO 80309 USA; ^4^ Materials Science and Engineering Program University of Colorado Boulder 4001 Discovery Dr, 613 UCB Boulder CO 80303 USA; ^5^ Department of Chemical and Biological Engineering University of Colorado Boulder 1111 Engineering Dr, 596 UCB Boulder CO 80309 USA; ^6^ Department of Earth and Environmental Sciences The University of Manchester Williamson Building Manchester M13 9PY UK; ^7^ Lancaster Environment Centre Lancaster University University Library Ave, Bailrigg Lancaster LA1 4YQ UK

**Keywords:** biodegradable electronics, decomposition, microbial activity, printed electronics, soil sensing

## Abstract

Soil health is one of the key factors in determining the sustainability of global agricultural systems and the stability of natural ecosystems. Microbial decomposition activity plays an important role in soil health; and gaining spatiotemporal insights into this attribute is critical for understanding soil function as well as for managing soils to ensure agricultural supply, stem biodiversity loss, and mitigate climate change. Here, a novel in situ electronic soil decomposition sensor that relies on the degradation of a printed conductive composite trace utilizing the biopolymer poly(3‐hydroxybutyrate‐*co*‐3‐hydroxyvalerate) as a binder is presented. This material responds selectively to microbially active environments with a continuously varying resistive signal that can be readily instrumented with low‐cost electronics to enable wide spatial distribution. In soil, a correlation between sensor response and intensity of microbial decomposition activity is observed and quantified by comparison with respiration rates over 14 days, showing that devices respond predictably to both static conditions and perturbations in general decomposition activity.

## Graphical abstract

A novel in situ electronic soil decomposition sensor that relies on the degradation of a printed conductive composite trace utilizing a biopolymer as a binder is presented. In compost tea and soil, a correlation between sensor response and microbial decomposition activity is observed and quantified, showing that devices respond predictably to both static conditions and perturbations in general decomposition activity.

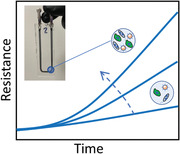

## 1 Introduction

Microbial activity is one of the key processes underlying the function of soils in natural and managed ecosystems and is tied to several ecological processes that ultimately affect the long term health of the planet.^[^ ^1^, ^2^, ^3^ ^]^ To date, the sensing of soil health has been limited to indirect field or laboratory measurements of microbial and enzymatic activity which can be time and labor intensive, and potentially biased by in‐lab conditions. This limits the ability of environmental scientists and growers to monitor and manage soil health.^[^ ^4^, ^5^ ^]^

### 1.1 Measuring Soil Health

The term “soil health” encompasses a range of different processes and metrics that include microbial diversity, activity, and the interactions of the biological and physical attributes of soils that determine important processes such as nutrient release and carbon decomposition. Collectively these processes play a major role in global agricultural productivity,^[^ ^6^ ^]^ have a major influence on the production and stabilization of greenhouse gases,^[^ ^7^ ^]^ and underpin societal functioning at large.

Standardized methods to assess the microbial diversity of soil typically involve the extraction of soil samples, transport to a laboratory, and the implementation of one or more of a selection of either biochemical techniques, such as plate counting, or microbiological techniques, such as polymerase chain reaction (PCR).^[^ ^1^, ^8^, ^9^ ^]^ While these are the best approaches to identifying the types of microbes in the soil and their functions, they do not quantify decomposition rates occurring in situ and do not properly capture spatiotemporal variability.^[^ ^8^, ^9^ ^]^ In addition, there is often a delay from when tests are performed to when decision‐makers, such as farmers, receive data.^[^ ^4^, ^5^ ^]^

The diversity of microbes in soils is important for broader agricultural or natural ecosystems because such organisms produce enzymes that degrade organic substrates, make nutrients available to plants, and produce carbon dioxide that is returned to the atmosphere following photosynthetic fixation in primary production.^[^ ^10^, ^11^ ^]^ There have been major advances in the measurement of soil enzymes, but like microbial diversity measures, most approaches are labor intensive and often require extensive laboratory analysis.^[^ ^12^ ^]^

Functional measures of decomposition also offer insights into soil health and there are several methods that can assess rates of decomposition or nutrient production/stabilization. These metrics involve measurements of the mass loss of specific materials that correlate well with the microbial dynamics noted above and vary across space and time in ways that are consistent with broader environmental factors, such as temperature and moisture.^[^ ^5^, ^13^ ^]^ Other functional metrics involve direct measurements of nutrient release and/or carbon dioxide production in soils.^[^ ^14^ ^]^ Mass loss methods include the use of cotton strips which can be a strong indicator of the decomposition rate of soil, especially for cellulose breakdown,^[^ ^15^ ^]^ and the Tea Bag Index (TBI),^[^ ^5^ ^]^ which utilizes the mass loss of a fast degrading labile material, green tea, and a slow degrading recalcitrant material, rooibos tea, in order to calculate a decomposition rate for a given soil without requiring the user to take multiple weight measurements over time.^[^ ^16^ ^]^ Unfortunately, measurements such as these take extended periods of time (weeks to months of material breakdown in the field) and are labor and time intensive.

For these reasons, there is interest in developing new approaches for the in‐situ measurement soil microbial activity sensing in real time. Existing approaches include a sensor that relies on comparing the respiration rate of pathogens in soil with that of microbes immobilized on a membrane.^[^ ^17^ ^]^ Another method correlates the current density of a microbial fuel cell with the microbial activity of the groundwater the cell is submerged in.^[^ ^18^ ^]^ In addition, a “microbial electrochemical technology” or “microbial electrochemical system,” which detects electrons emitted or accepted by microbes as they digest organic matter,^[^ ^19^, ^20^ ^]^ has shown promising results for in situ soil microbial activity sensing using a relatively simple apparatus.^[^ ^21^ ^]^ Despite these novel technologies, a direct, real‐time, in situ measure of microbial processes remains elusive.

### 1.2 Biodegradable Electronics

There is significant ongoing research and development into biodegradable electronics, primarily for biomedical applications, that could be adapted for use in soils. Typically, failure and subsequent degradation is a secondary functional requirement in biodegradable sensors, falling behind the primary sensing function. In some cases, the transience of certain components has been used to sense food spoilage, for example via change in the response of a degradable radio‐frequency identification (RFID) antenna. This can result from the change in dielectric properties caused by the spoilage or ripening of the medium underneath,^[^ ^22^ ^]^ the development of a biofilm on top of the antenna, or the degradation of a polyaniline encapsulant by chemical gasses associated with spoilage.^[^ ^23^ ^]^ The concept of a degradation sensor using poly(lactic acid) (PLA) andcarbon nanotube nanocomposites was proposed by Mai, et al., wherein the resistivity decreases as a result of the nonenzymatic hydrolysis of amorphous regions and subsequent recrystallization of the remaining PLA.^[^ ^24^ ^]^ Samples are desiccated before resistance measurements are taken, precluding its use as an in situ sensor without further development. Printed conductive traces comprising a PLA binder have been shown to degrade rapidly in an enzyme solution while staying relatively stable in water^[^ ^25^ ^]^ and water‐soluble traces encapsulated in wax blends have been shown to act as fuses whose failure in soil is easily discernible from failure in wet sand or aqueous solutions.^[^ ^26^ ^]^

**Figure 1:**
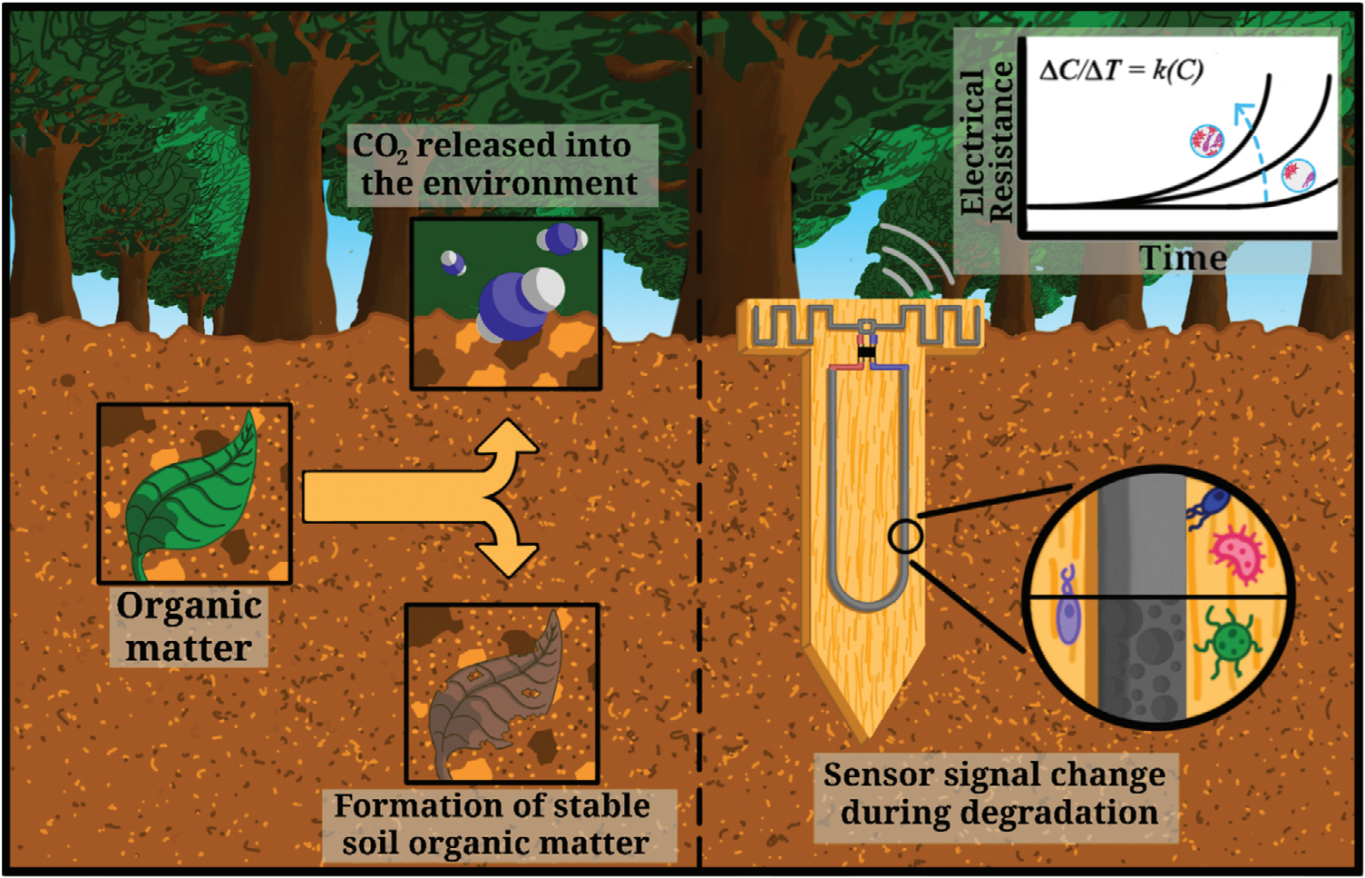
Concept of a decomposition sensor where the rate of erosion of a biodegradable conductive trace correlates with the microbial activity in the soil.

As with methods such as the TBI, monitoring the decomposition of certain materials can shed light on the microbial activity of soil. In that same vein, here we propose that printed biodegradable conductors, comprising the soil‐degradable polymer, poly(3‐hydroxybutyrate‐*co*‐3‐hydroxyvalerate) (PHBV) as a binder, can act as in situ microbial activity or decomposition sensors (see **Figure** **1**) by monitoring its conductivity with low‐cost wireless electronics. We compare the resistance increase of these conductive traces with microbial respiration rates and show that a correlation can be drawn within 6 days of incubation.

## 2 Results and Discussion

### 2.1 Ink Development and Characterization

**Figure 2:**
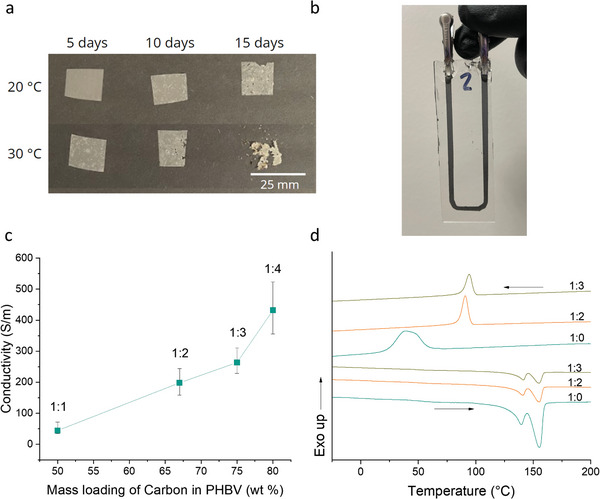
Development of a PHBV‐based biodegradable ink. a) Degradation of stock PHBV films at 20 and 30 °C. b) PHBV:C trace printed and prepared for degradation testing. c) Plot of conductivity of printed traces as a function of mass loading of C. d) DSC thermogram of first heating curves (→) and first cooling curves (←) of stock PHBV films (1:0) and PHBV:C traces.

Biodegradable conductive materials typically comprise conductive particles, such as tungsten, molybdenum, or zinc, held together by a binder, such as a polymer,^[^ ^25^, ^27^, ^28^, ^29^ ^]^ polysaccharide,^[^ ^30^ ^]^ or wax,^[^ ^31^ ^]^ that degrades due to a certain stimulus. As many of these prior studies focused on biomedical applications, these stimuli are often the presence of water or buffer that induces either hydrolysis or dissolution of the binder. While a PLA binder has been shown to be water stable but respond to enzymatic hydrolysis,^[^ ^25^ ^]^ the degradation of PLA in soil is too slow for utility as a decomposition sensor. Here, we have selected the bio‐derived polymer PHBV, a polyhydroxyalkanoate (PHA), as a binder due to the relatively rapid degradation of PHAs in both aerobic^[^ ^32^, ^33^, ^34^ ^]^ and anaerobic soils.^[^ ^33^ ^]^ PHBV is degraded by several bacteria and fungi found in the soil via extracellular PHB depolymerase enzymes.^[^ ^33^, ^34^ ^]^ Increasing temperature between 15 and 40 °C has been shown to greatly affect degradation rates of PHBV films, due to the increased microbial activity.^[^ ^32^, ^35^, ^36^, ^37^, ^38^ ^]^ The PHBV (8% hydroxyvalerate content) films that were selected as the feedstock for our binder were tested as‐purchased in potting soil at room temperature and 30 °C, both to demonstrate soil degradability of the binder material and to demonstrate the effect of elevated temperature. As shown in **Figure** **2**a, temperature plays a significant role in accelerating the degradation. The development of holes during the degradation of stock 0.025 mm thick films, suggests that surface erosion is occurring.^[^ ^39^, ^40^, ^41^ ^]^ Previous literature has shown that PHB maintains molecular mass during biodegradation at temperatures below 40 °C,^[^ ^32^ ^]^ implying that PHB surface erodes rather than volume erodes.^[^ ^42^, ^43^ ^]^

PHBV films were dissolved in chloroform at a concentration of 110 mg mL^−1^ and different quantities of 10 µm carbon flake (C) were added to form printable conductive inks, which were then stencil‐printed onto glass substrates. Carbon was selected due to its chemical inertness. Soluble metals such as Mo and W were not used since oxidative their degradation could lead to changes in resistance that are unrelated to microbial activity.^[^ ^25^ ^]^ In addition, oxides formed by such metals often have a cytotoxic effect,^[^ ^44^, ^45^ ^]^ precluding their use in a soil microbial activity sensor. Additionally, solid carbon can be left in the soil,^[^ ^46^ ^]^ eliminating the need for retrieval once such a trace is integrated into a fully biodegradable or bioinert sensor package. Glass was used as an inert substrate to isolate the behavior of the PHBV‐C composite only, without interaction from a biodegradable substrate. An example of such a trace as printed and prepared as a decomposition sensor is shown in Figure 2b. Conductivity in composites such as these develops as a result of percolation pathways formed by conductive particles; and there is a percolation limit after which increasing the ratio of particles does not yield an improvement in conductivity.^[^ ^28^, ^47^, ^48^ ^]^ As shown in Figure 2c, we did not approach an obvious percolation limit with the weight ratios tested due to issues related to printability at higher weight ratios of C.

The crystallinity of PHBV (and of biodegradable polymers in general) affects its rate of degradation due to the fact that amorphous regions are more vulnerable to enzymatic hydrolysis.^[^ ^49^, ^50^, ^51^ ^]^ While PHBV is a highly crystalline polymer, increasing the  hydroxyvalerate content decreases crystallinity, and consequently accelerates PHBV's rate of degradation.^[^ ^49^, ^50^, ^52^ ^]^ The first heating curves from differential scanning calorimetry showed that the addition of carbon at 2:1 and 3:1 by weight had no significant effect on melting temperature or heats of fusion of the PHBV in the binder (once normalized for weight ratio) as compared to the commercial film (see Figure 2d for curves; and Table S1 for enthalpy values, Supporting Information), implying that confinement by carbon particles did not affect crystallinity.^[^ ^51^, ^53^, ^54^ ^]^ However, the crystallization temperature (T_C_) was significantly increased by the addition of carbon, likely due to the carbon flake acting as a nucleating agent, which tends to significantly aid in PHBV crystallization.^[^ ^55^, ^56^, ^57^ ^]^ This increase in T_C_ suggests a decrease in chain mobility, which could in turn reduce its rate of degradation.^[^ ^58^, ^59^, ^60^ ^]^

### 2.2 Degradation Studies in Compost Tea

**Figure 3:**
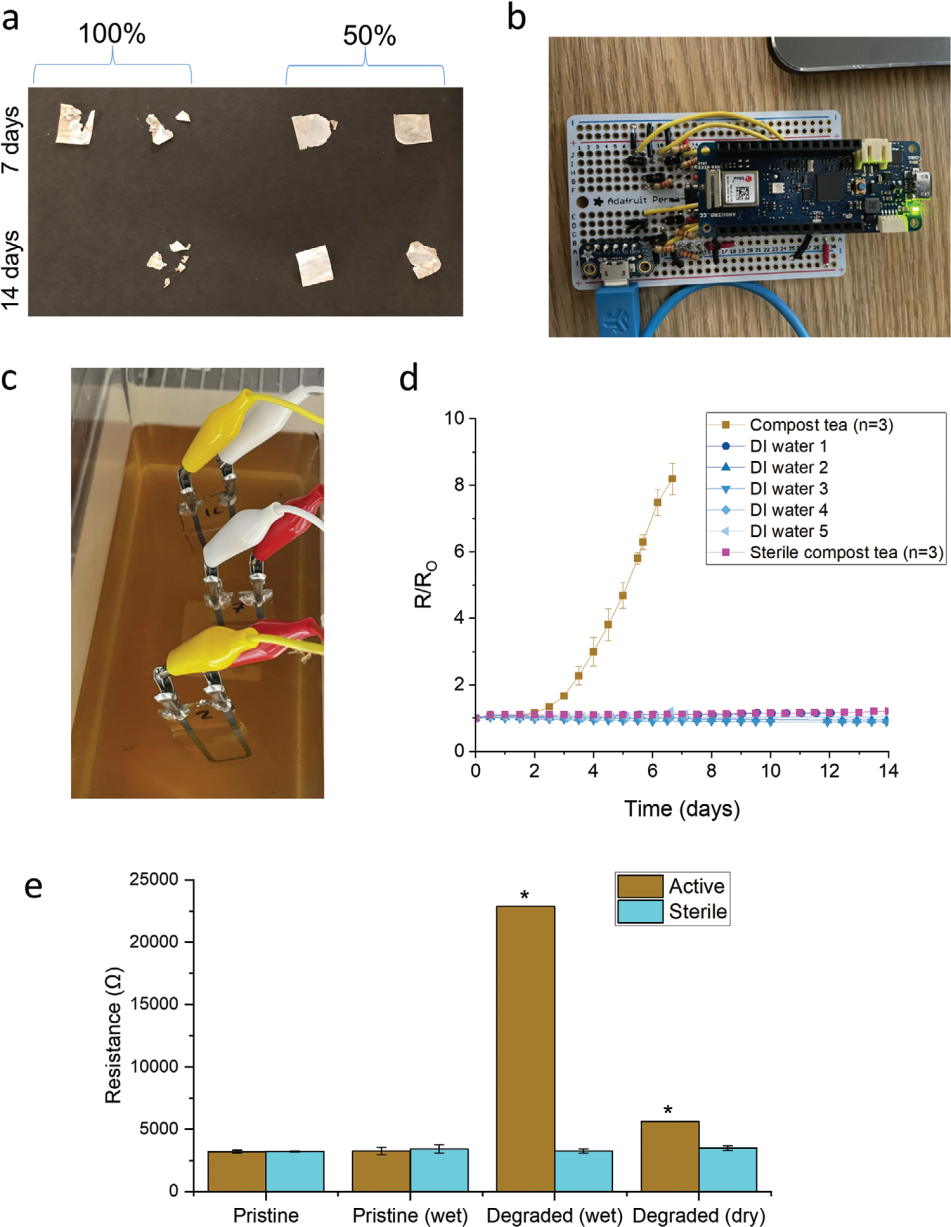
Sensor evaluation in compost tea. a) Degradation of as‐purchased 0.025 mm PHBV films in 30 °C 100% and 50% compost tea. b) WiFi Arduino‐based data acquisition circuit. c) Photograph of PHBV‐C traces in 35 °C 100% compost tea. d) Normalized resistance of PHBV‐C conductive traces in compost tea, sterilized compost tea, and DI water. e) Plot of resistances obtained from the slope of the *I*–*V* curve for sensors in active and sterilized compost tea in different wet and dry conditions (pristine, in liquid for 1.5 h, incubated in liquid for 7 days, and then dried in ambient conditions for 2 days). Star indicates *n* = 2 due to disintegration of one trace during measurement as compared to *n* = 3 for other data points.

Previously, we showed that the conductivity achieved by percolation pathways formed in a biodegradable conductive ink can be rapidly broken down by enzymatic activity in a liquid medium.^[^ ^25^ ^]^ In that study, the resistance of the trace increased at a much faster rate due to enzymatic attack and conductive particles falling out of the matrix, than due to simple swelling in water. Hence, it is conceivable that erosion of the biodegradable binder in a printed conductive trace submerged in a microbially active liquid medium would yield a detectable increase in resistance. In order to investigate the effect of microbially active liquid media on the PHBV‐C conductive composite, printed traces were tested in “worm tea,” also known as “microbe brew” or “compost tea.”^[^ ^61^ ^]^ As shown qualitatively in **Figure** **3**a, as‐purchased PHBV film disintegrates rapidly in 100% compost tea with degradation significantly affected by dilution (mass loss data can be found in Table S2, Supporting Information).

Ink comprising a 3:1 ratio by weight of 10 µm C flake and PHBV was stencil printed onto glass slides to form traces 3 mm wide and ≈0.06 mm thick. These samples were submerged in 100% compost tea and incubated at 35 °C for 7 days (Figure 3d). Resistance was monitored with an Arduino‐based circuit that collects and logs resistance measurements wirelessly (see Figure 3b for photograph and Figure S1 for circuit diagram, Supporting Information). The experiment was repeated with sterilized compost tea and deionized (DI) water. Figure 3c shows the plot of resistance change over time of these traces, demonstrating very rapid failure of the traces in the compost tea with minimal change of resistance of traces in sterilized tea (similar to DI water). Therefore, it can be concluded that the failure of the traces, and the degradation of the films are likely due in large part to microbial activity and not due only to hydrolytic activity from other properties of the compost tea. This is to be expected as prior studies have shown the mass loss of PHBV due to hydrolysis from salinity in a buffer system or seawater is on a much slower scale.^[^ ^49^, ^62^ ^]^ In order to rule out other possible causes of sensor failure in microbially active compost tea, such as failures at contact points, similar nonbiodegradable traces were printed from an ink comprising a 3:1 ratio by weight of C flake and poly(methyl methacrylate) (PMMA). These traces showed no significant increase in resistance over a span of 18 days in 35 °C compost tea (see Figure S2, Supporting Information), implying that resistance changes of PHBV‐C sensors in microbially active compost tea are not likely due to the degradation of other components in the signal chain, and are due to the use of the biodegradable PHBV binder.

Several tests were conducted to investigate the mechanism of degradation which causes a change in sensor resistance. To evaluate geometric change, confocal microscope measurements of dried PHBV‐C traces before and after degradation for 7 days in a beaker of compost tea at 35 °C were taken and revealed no significant change in cross‐sectional area (see Figure S3 for thickness profiles and comparison, Supporting Information). Additionally, scanning electron micrographs (SEM) taken of PHBV‐C and PMMA‐C pristine and incubated traces revealed no obvious internal structural changes (see Figure S4, Supporting Information). To test for the presence of cracks in the traces, current–voltage (*I*–*V*) sweeps were conducted on different lengths of a trace before and after degradation for 7 days (and subsequent drying). Pristine traces were Ohmic throughout before and after, with length‐independent resistivity (at these scales), suggesting that the observed changes in bulk resistivity are not due to large‐scale removal of conductive trace material at any point (see Figure S5 for plot, Supporting Information).

Typically, in hydrolytic environments, the degradation kinetics of a polymer transitions from surface‐dominant erosion to volume‐ (or bulk‐) dominant erosion below a certain critical thickness.^[^ ^43^ ^]^ While most literature suggests that, at practical temperatures, PHBV samples surface erode (no observed change in M_W_) from microbial consumption,^[^ ^32^, ^39^, ^41^, ^51^, ^54^ ^]^ nanofibers of PHB have been shown to decrease in M_W_ as a result of degradation in soil, implying volume erosion.^[^ ^34^ ^]^ The average thickness of a polymer in a conductive composite, such as the PHBV‐C composites presented here, is typically in the nanometer scale to allow percolation pathways to occur through the filler material.^[^ ^25^, ^28^, ^63^, ^64^ ^]^ Therefore, the binder in these traces are likely volume eroding, explaining why there are no apparent bulk physical differences between dried samples before and after degradation. However, as polymers biodegrade due to microbial activity, their uptake of water tends to increase,^[^ ^65^ ^]^ implying that sensors that are subject to a microbially active environment would swell compared to those in sterile environments, which could subsequently lead to an increase in interparticle spacing, thereby causing an increase in resistance.^[^ ^25^, ^28^ ^]^ To verify this, *I–V* sweeps were conducted on both dry and wet sensors before and after incubation. Figure 3e, plotting the resulting sensor resistances, shows a significant difference between wet and dry degraded sensors as compared with those placed in sterilized tea. In addition, laser confocal microscopy revealed that a trace subject to incubation in microbially active compost tea for 7 days was 28% thicker when wet than after drying. In comparison, a trace subject to incubation in sterilized compost tea was only 1% thicker. These data further suggest that biodegradation of the PHBV binder leads to increased swelling in wet environments, which in turn leads to the observed increase in resistance (see Figure S6, Supporting Information).

### 2.3 Soil Degradation Studies

**Figure 4:**
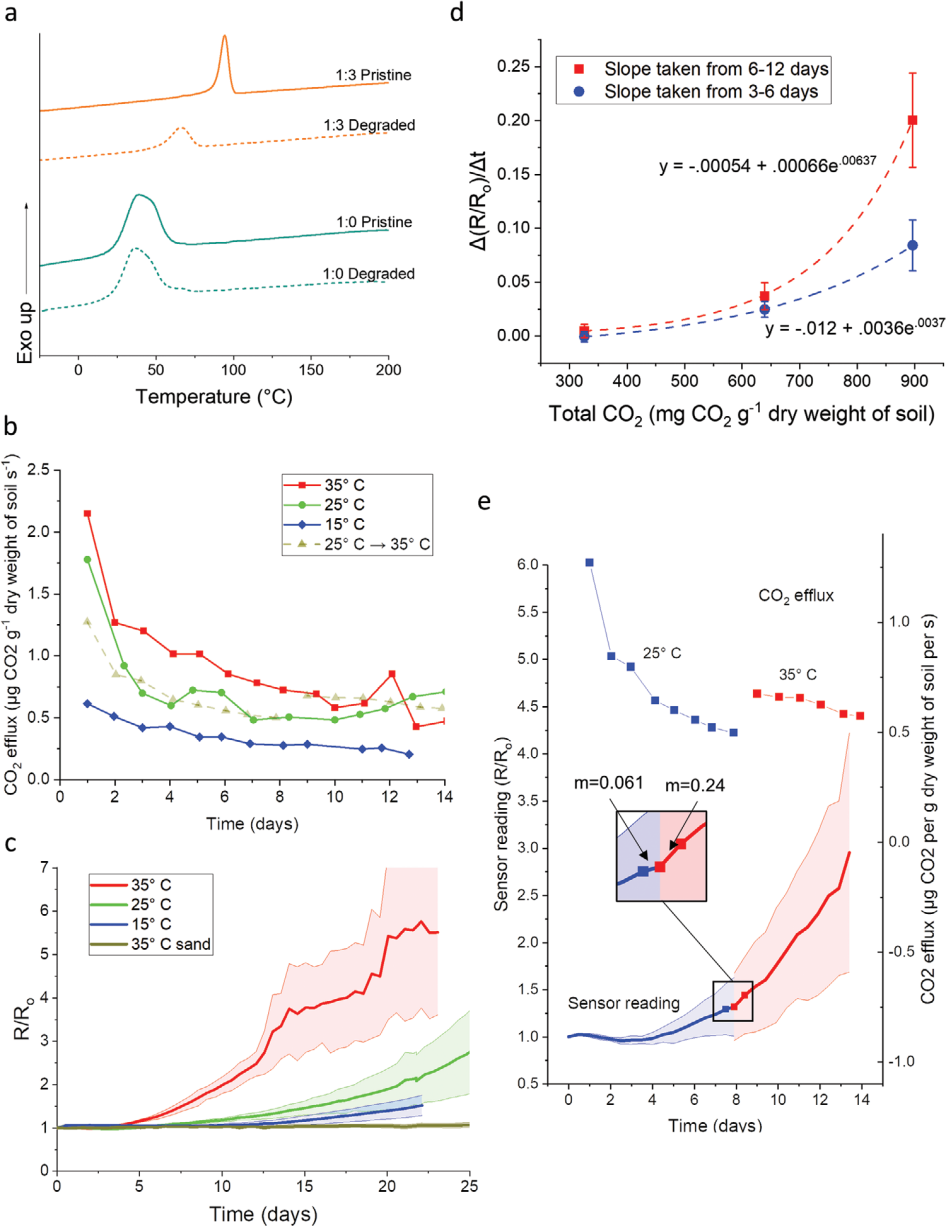
Sensor evaluation in soil. a) DSC cooling curves of PHBV (1:0) and PHBV‐C (1:3) before and after degradation in soil for 6 days at 35 °C. b) Plot of CO_2_ efflux from soils. c) Plot of normalized resistances of PHBV:C (1:3) conductive traces in soil. d) Plot of slopes obtained from linear regression of points from plot b) from 3 to 6 days and 6 to 12 days versus a total efflux in 12 days. d) Plot of CO_2_ efflux from soil and normalized resistances of PHBV:C (1:3) conductive traces in soil, where the soil temperature was increased from 25 to 35 °C after 8 days.

As discussed in Section 3.1, PHBV degrades rapidly in soil, and therefore is an excellent candidate for a binder in the development of a printed soil decomposition sensor. Since damage due to removal of traces from soil precludes the use of techniques such as cross‐sectional measurements made with a confocal microscope, differential scanning calorimetry (DSC) was performed on a 3:1 composite degraded in 30 °C saturated potting soil for 6 days (see **Figure** **4**a). The first heating run showed no significant effect on the melting peaks (see Figure S7, Supporting Information), implying crystallinity of retrieved pieces had not changed.^[^ ^51^, ^53^, ^54^ ^]^ However, the T_C_ of 66.4 °C was significantly less than that of the pristine trace, which suggests that molecular weight (*M*_W_) was potentially reduced during degradation, thereby implying a volume erosion process^[^ ^66^ ^]^ (note that melting temperature only decreases significantly if PHBV's *M*_W_ drops below 60 000 g mol^−1^).^[^ ^60^, ^67^, ^68^ ^]^ Degradation of a stock film under the same conditions did not show significant change in T_C_. This correlates to qualitative observations of the degraded films, shown in Figures 2a and 3a, which developed holes that are characteristic of surface erosion.

To correlate the failure behavior of PHBV‐C traces with soil microbial activity, CO_2_ efflux measurements^[^ ^21^, ^69^ ^]^ were performed alongside degradation tests. Here, we use temperature to vary soil microbial activity. By a factor known as the Q10 value, microbial activity increases with temperature between 15 and 35 °C. This range was selected since temperatures above this range would likely cause decline in activity due to enzyme denaturing, and below this range would cause degradation to slow down to rates too low for practical experimentation.^[^ ^35^, ^36^, ^37^, ^38^, ^69^, ^70^, ^71^ ^]^ Traces (*n* = 3 per treatment) were buried in collected soil kept at 35, 25, and 15 °C, with heat‐sterilized fine sand at 35 °C as a control. Both the soil and sand were watered (with tap water for the former and DI water for the latter) to field capacity and rewatered regularly to keep VWC constant within 5%. To confirm that varying temperature varied the microbial activity of soil, microbial respiration was regularly measured from the media by temporarily sealing the container and measuring CO_2_ for approximately a half hour. As expected by the Q10 rule,^[^ ^69^, ^70^, ^71^ ^]^ soil microbial activity varied with temperature, with values for daily efflux for the three temperatures plotted with solid lines in Figure 4b. As expected, sand had no positive CO_2_ efflux, implying negligible microbial activity. Due to there being a limited availability of carbon (in the soil and to a much lesser extent, the PHBV binder in the sensor), microbial activity in soil decreased as the experiment progressed.^[^ ^72^ ^]^

As demonstrated in Figure 2a and in prior literature, the rate of degradation of PHBV increases with increased temperatures, due to increased microbial activity.^[^ ^32^, ^35^, ^36^, ^37^, ^69^, ^70^, ^71^ ^]^ The resistance change of the PHBV‐C traces in incubated soils of different temperatures, measured using the same Arduino‐based circuit mentioned in Section 3.2, is plotted in Figure 4c, where it is apparent that failure rates of traces vary at different temperatures. At 14 days, the sensors in 15, 25, and 35 °C soil showed an average 14%, 43%, and 314% increase in resistance, respectively. In 35 °C sand (used as a control experiment), sensors exhibited an average 4% increase in resistance in 14 days and a 13% increase in 30 days. This small change is due to the lack of microbial activity to degrade the PHBV binder. These data suggest that the PHBV‐C traces are degrading due to the microbial activity in soil and not just due to elevated temperature in aqueous media. Again, SEM revealed no obvious structural changes between pristine and degraded traces, suggesting a similar mode of degradation as discussed in Section 2.3. The error bars are likely the result of natural variations in soil systems, as well as variability in the fabrication of the sensor packages themselves. Performing a linear regression for the normalized resistance values from data between 3–6 days and 6–12 days and then comparing it to an estimated total CO_2_ released by each system in 12 days yields the sensor response curve shown in Figure 4d. It is apparent in this figure that, while a correlation between microbial activity and sensor readings can be drawn in 6 days, 12 days provides a clearer picture. Multivariable regression was conducted on sensor response (slopes taken between 6 and 12 days) as a function of both total CO_2_ released (*p* = 0.02) and initial resistance (*p* = 0.73), further emphasizing the correlation between microbial activity and sensor response and ruling out the effects of sensor‐to‐sensor variability.

In order to investigate PHBV‐C sensor response to dynamic changes in microbial activity, incubation temperature was started at 25 °C and was ramped up to 35 °C on day 8. As can be seen with the dotted lines in Figure 4b, ramping up the temperature moved the CO_2_ efflux curve from initially tracking the previous 25 °C curve from to tracking the 35 °C curve, confirming that microbial activity was indeed increased. The resulting plot of normalized resistance of PHBV‐C sensors overlaid with the CO_2_  efflux plot, shown in Figure 4e, shows a local fourfold increase in slope that occurs as a result of the increased microbial activity, indicating that these devices are able to detect an increase in decomposition activity during their service life.

The soil experiments described above (and displayed in Figure 4) were carried out at constant soil moisture content in order to provide controlled experimental conditions to isolate the impact of soil microbial activity on sensor response. Experiments were additionally carried out with varying moisture levels by subjecting similar sensors to potting soil and a sand control that were saturated with water and then allowed to dry out before weekly resaturation with water (Figure S8, Supporting Information). Under these conditions, a similar response to temperature induced microbial activity changes is observed (increasing slope of relative resistance with increasing temperature), as is observed when soil moisture content is kept constant.

## 3 Conclusion

In this work, we show that PHBV‐C conductive traces can be used as decomposition sensors in liquid media or soil, where the failure rate over time correlates to the microbial decomposition capability of the medium. These sensors show a reliable and selective response for degraders of PHBV in liquid solutions of compost tea and demonstrate a correlation between microbial respiration and sensor response in soils kept at field capacity. Volume erosion of the biodegradable PHBV binder is shown to be the likely cause of resistance change as it leads to increased swelling in the degraded traces compared with controls. As PHB and PHBV depolymerase enzymes are produced by both fungal and bacterial microbes common in soils of many types, these sensors should enable correlation with the overall decomposition activity of a given soil or liquid. As demonstrated with Arduino‐based wireless data acquisition circuits, these resistive sensors can be easily addressed, enabling straightforward integration with low‐cost, off‐the‐shelf, electronics, enabling distributed in situ quantitative monitoring of soil microbial activity. In addition, print‐based fabrication allows for facile adaptation to mass manufacturing. These combined properties offer a novel, practical, and low‐cost approach to gaining insights into soil microbial functioning in situ and at scale, opening possibilities for expanding our scientific understanding and improving our capacity to manage soils as a critical resource. Following on from these studies, additional materials could be explored as binders in similar configurations to gain a better understanding of the activity of specific microbes and enzymes, and analytical methods such as PCR can be used to characterize microbial populations responsible for degradation. Ultimately, these printed, soil compatible sensors could enable improved characterization of the soil microbiome and its carbon degradation compatibility, in order to enhance soil health and potentially improve soil carbon accounting approaches.

## 4 Experimental Section

### Ink Formulation, Printing, and Electrical Characterization

PHBV film (*Goodfellow*, 8% valerate content, 0.025 mm or 0.01 mm thick) was dissolved in chloroform at a concentration of 110 mg mL^−1^ in a vial on a hotplate at 45 °C. Carbon (C) flake (*Graphene Supermarket*, 10 µm) was added to the solution and mixed by manually shaking and with a vortex mixer. Traces used for electrical characterization (conductivity measurements) were fabricated by first adhering double sided tape on a 0.25 mm Mylar sheet and then laser cutting stencils. Stencils were then attached to glass microscope slides. Stencils for traces for continuous sensor studies were fabricated by adhering two layers of Kapton tape directly on glass microscope slides and then laser cutting the required pattern (the resulting stencil thickness is 0.1 mm). Traces were printed by hand using a razor blade^[^ ^28^ ^]^ to spread ink across the stencil (at an approximate blade speed of 0.075 m s^−1^ and an average maximum force of 60 g). Stencils were peeled off, traces were placed on a hotplate to dry, and then stored at room temperature. The thickness of two to three locations on ten traces were measured using a micrometer, yielding an average thickness of 0.062 mm (SD = 0.012). Stainless steel terminal clips were attached to the trace using silver epoxy (*MG Chemicals* 8331D). Once the silver epoxy was cured, these joints were then covered in epoxy (*Gorilla*). Volume resistivity was measured on the traces using a multimeter, calipers, and micrometer. The mass ratio used for continuous sensor studies was 1:3 PHBV:C. A nonbiodegradable version of this ink was fabricated by dissolving PMMA (Sigma‐Aldrich, *M*_W_ ≈350 000) in anisole at a concentration of 110 mg mL^−1^ and then mixing in carbon flake at a ratio of 1:3 PMMA:C.

### Sensor Instrumentation

For continuous sensor experiments, resistance was monitored with the use of a custom WiFi‐based circuit comprising an Arduino MKR 1010, an AS358P operational amplifier integrated circuit (op amp) chip, and voltage dividers with 2.2 kΩ reference resistors. The Arduino and IC chip were each fed power from a USB breakout board. The circuit is designed to supply a stable voltage without using the Arduino or loading the source through an op amp and an input. The Arduino can control when the circuit turns on so as not to constantly use power. Each circuit was designed to monitor 6 resistances simultaneously. Every 12 h, 5 measurements were taken over the span of 5 s and sent wirelessly to a Google Sheet. To demonstrate the accuracy of resistance readings, commercial resistors were tested and compared to readings from a multimeter. The plot, shown in Figure S9 (Supporting Information), demonstrates sufficient accuracy in the region of interest (1600–10000 Ω).

### Degradation Studies

For studies conducted in compost tea, *Microbe Brew* was purchased from Eco‐Cycle in Boulder County, CO. According to Boulder County Eco‐Cycle, *Microbe Brew* compost tea is produced by capturing the runoff of vermicompost and then increasing microbial activity through an additional fermentation step.^[^ ^73^ ^]^ Compost tea was used as‐is within the expiration date prescribed by the producer. Qualitative and quantitative mass loss studies were conducted by incubating as‐purchased 0.025 mm films in either 100% compost tea or 50%, diluted with reverse osmosis filtered water at 30 °C. Remaining pieces were dried at 45 °C dynamic vacuum for at least 24 h before photographing and weighing. Three individually monitored PHBV:C traces (continuous sensors) were submerged in a container of compost tea which was incubated at 35 °C for a week. For the control, three similar traces were submerged in the same setup with sterilized compost tea (which was autoclaved for 50 min and stored at 4 °C for three days until use). Additional controls were run in DI water, both in a similar container and in separate beakers.

Additional PHBV:C traces were incubated in beakers of compost tea for 7 days at 35 °C for electrical characterization. For the first trace, a multimeter was used to measure the resistance of three sections and laser confocal microscopy was used to measure trace thickness and width before and after incubation. On a separate trace, IV sweeps were conducted at different lengths before and after incubation using a Keithley 2636a System SourceMeter combined with a custom LabView program. All other IV sweeps were conducted using the same device and program.

Continuous sensor studies in soil were conducted using soil collected from the top 25 cm of a field site in Boulder County, Colorado, USA, (40°02″26.9“N 105°08”34.4″W). Collected soil was sieved through a 2 mm sieve and rocks and large litter were removed. Analysis of soil from the same site collected at an earlier date showed a dissolved organic carbon (DOC) content of 56.0 mg kg^−1^ and a pH of 7.75.  Sieved soil was stored at 4 °C until time of use. 20 cm wide by 10 cm deep by 20 cm high plastic storage containers were prepared by drilling holes in the side for wire passthrough (which were then sealed) and holes in the lid for ventilation. Sensors were placed in dry media before the media was watered to approximate field capacity. For each soil treatment (*n* = 3), 1 kg of dry soil was watered with 300 g of tap water (30% volumetric water content [VWC], ≈1.3 g cm^−3^ as the bulk density of dry soil). Fine sand (*Sandtastik* Play Sand) was used for the control group. 1.3 kg of sand was watered with 300 g of deionized (DI) water. The water content of the media was maintained within 5% VWC by manual watering. On days that CO_2_ measurements were taken, the lid with ventilation holes was removed and replaced by a sealed lid with a cutout for a CO_2_ sensor (*Vernier GoDirect* CO_2_ Gas Sensor). Measurements, which were in units of PPM CO_2_, were taken for approximately a half hour. To calculate CO_2_ efflux, a linear regression was performed on the latter linear portion of the reading (inconsistencies in the beginning of the reading were attributed to either inherent transience or sensor error, as confirmed by the manufacturer). The efflux, in µg CO_2_ per gram dry weight of soil per second was calculated using a headroom volume of 2362 mL and the conversion from PPM to mg mL^−1^ for a given temperature (0.00186 mg mL^−1^ at 15 °C, 0.00180 at 25 °C, and 0.00174 at 35 °C).^[^ ^74^ ^]^ Total CO_2_ output was calculated by taking the Riemann integral from efflux plots. Slopes of R/Ro versus time plots were taken from 3 to 6 days and 6 to 12 using linear regression. Multivariable regression was conducted on the slope between 6 and 12 days as a function of total CO_2_ and initial sensor resistance to rule out the effect of initial resistance.

In all studies, containers along with the sensor and data acquisition circuits were placed in either an incubator for experiments above room temperature or in a refrigerator with a thermostatic controller for 15 °C experiments.

The study to assess the effect of weekly rewatering was conducted with the same traces as used for electrical characterization, using either consumer potting mix (Miracle Gro) or sand (Quickrete Play Sand) as media. Containers were prepared by modifying 2.5 in. acrylic cubes with lids (Dayanee). A drain hole was pierced on the bottom of the cube and a similarly sized vent/watering hole on the lid. 3 slots were cut on the lid for the sensors. Cubes were filled with either media. Sensors were placed through the slots and the media was saturated with tap water and resaturated weekly.

### Differential Scanning Calorimetry

Thermal characterization was carried out on cast samples using DSC (Q2000, TA Instruments). Samples were equilibrated at −50 °C, heated to 250 °C at 10 °C min^−1^, and then cooled back to −50 °C at −10 °C min^−1^. The soil degradation test for the sake of DSC was conducted in fully saturated consumer potting mix (*Miracle Gro*).

### Scanning Electron Microscopy

SEM was conducted on printed conductive traces using a Hitachi SU3500 scanning electron microscope, in order to evaluate differences between printed traces before and after degradation. Samples were prepared by sectioning of the glass slides and included printed traces. Samples comparing pristine and compost tea‐incubated traces were first submerged in liquid nitrogen prior to sectioning. Samples were sputter coated with platinum before imaging.

### Laser Confocal Microscopy

Surface profile measurements were taken using a Keyence VK X‐1100 profilometer. For volume measurements of dried traces before and after degradation, an area average was taken. For thickness measurements, an average of 10 profiles were used to calculate each thickness measurement and 3 measurements were taken along the trace. Measurements of wet traces were conducted by removing the trace from compost tea and removing surface droplets with a Kimwipe laboratory wipe. Traces were subsequently dried for at least 24 h in ambient conditions for dry measurements.

## Conflict of Interest

The authors declare no conflict of interest.

## Supporting information

Supporting Information

## Data Availability

The data that support the findings of this study are available from the corresponding author upon reasonable request.
