# Supplementary material for: A Transient Printed Soil Decomposition Sensor Based on a Biopolymer Composite Conductor
Source: Adv Sci (Weinh). 2022 Dec 11;10(5):2205785. doi: 10.1002/advs.202205785 (PMC9929122; doi:10.1002/advs.202205785)
Supplement: Supplementary file 1 — Supporting Information [file ADVS-10-2205785-s001.pdf]

ADVANCED SCIENCE
Open Access

Supporting Information

for *Adv. Sci.*, DOI 10.1002/advs.202205785

A Transient Printed Soil Decomposition Sensor Based on a Biopolymer Composite Conductor

*Madhur Atreya, Stacie Desousa, John-Baptist Kauzya, Evan Williams, Austin Hayes, Karan Dikshit, Jenna Nielson, Abigail Palmgren, Sara Khorchidian, Shangshi Liu, Anupam Gopalakrishnan, Eloise Bihar, Carson J. Bruns, Richard Bardgett, John N. Quinton, Jessica Davies, Jason C. Neff and Gregory L. Whiting**

Table S1: Enthalpy values* for as-fabricated PHBV film (1:0) and conductive traces PHBV:C (ratio by mass) in J/g.

	1:0	1:2	1:3
Pristine	77.381	79.086	84.216
Degraded	82.38	77.496	85.392

* values normalized for actual amount of PHBV in sample

Table S2: Degradation of as-purchased 0.025 mm PHBV film in compost tea

Sample	Compost tea concentration	Time in incubator	Initial dry mass of sample (mg)	Final dry mass of sample (mg)	% mass loss
**1**	100%	7 days	6.86	4.32	37%
**2**	100%	7 days	6.40	2.21	65%
**3**	100%	14 days	6.04	n/a	n/a
**4**	100%	14 days	6.87	1.83	73%
**5**	50%	7 days	6.64	3.89	41%
**6**	50%	7 days	6.00	5.47	8%
**7**	50%	14 days	6.31	5.43	14%
**8**	50%	14 days	6.81	2.94	57%

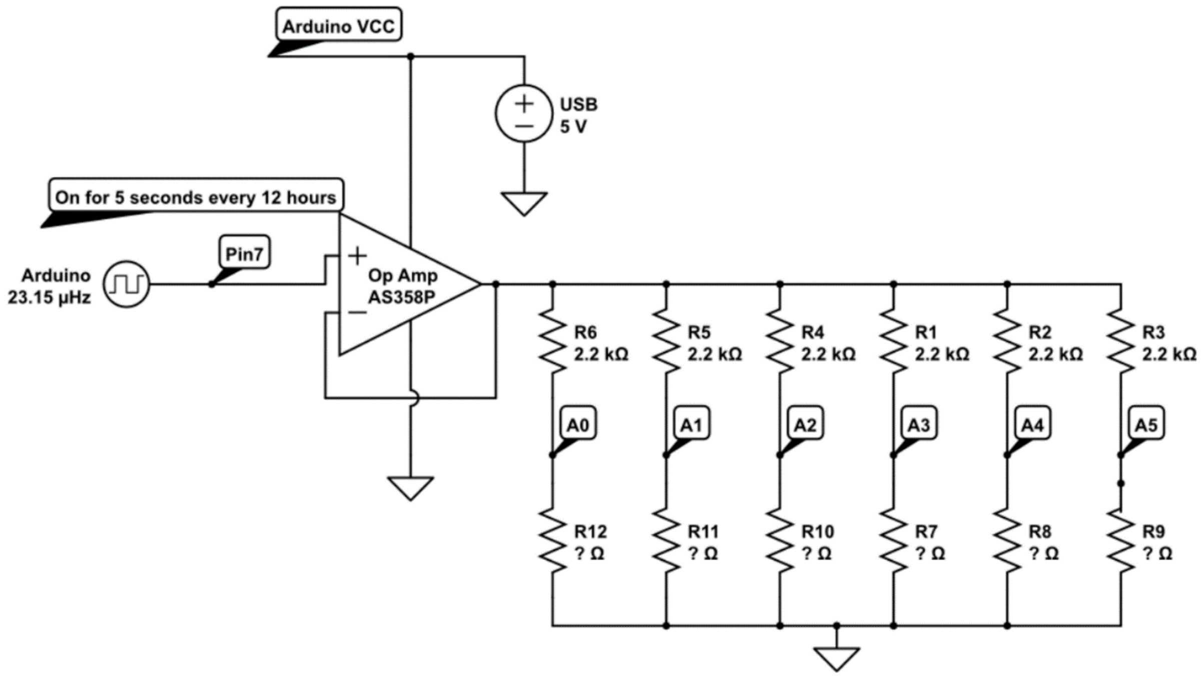

Figure S1: Circuit diagram of Arduino-based data acquisition circuit. (Created using CircuitLab).

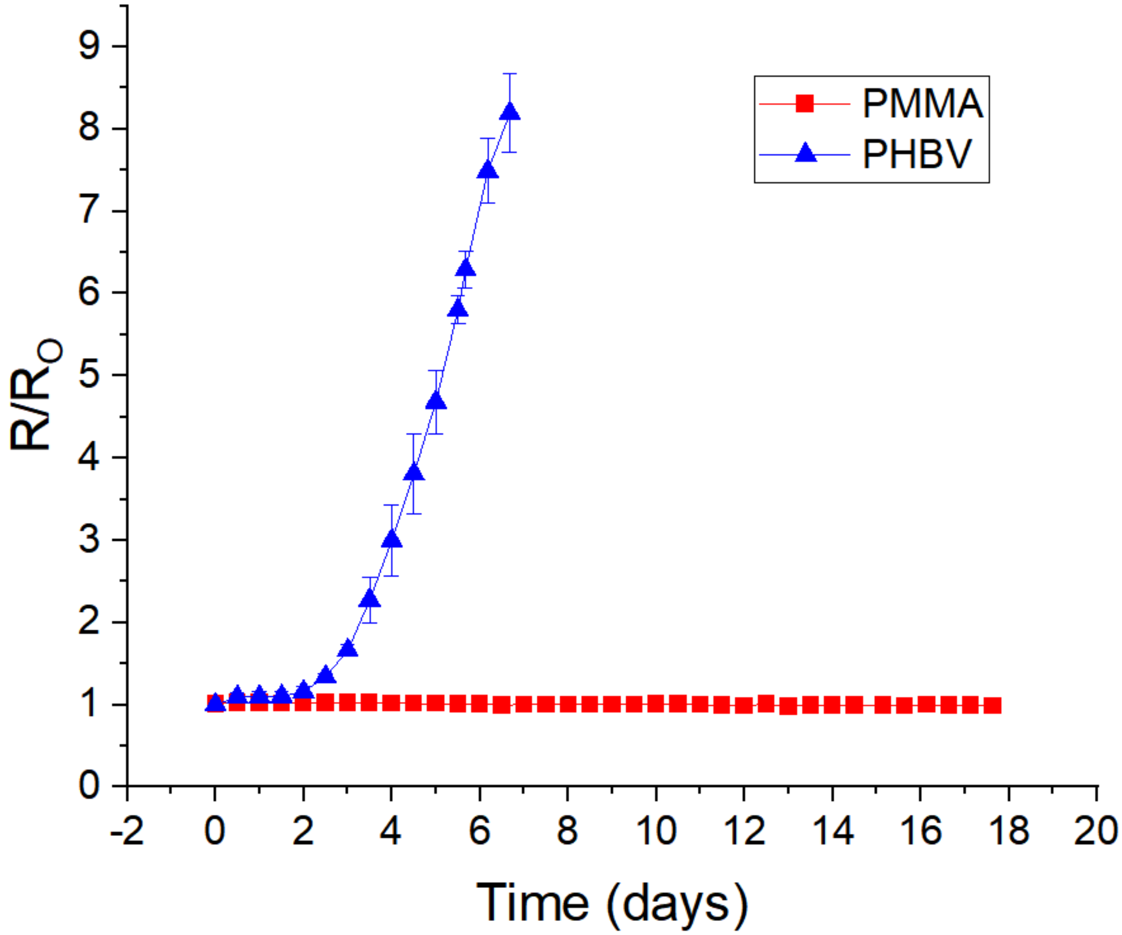

Figure S2: Normalized resistance of PHBV-C and PMMA conductive traces in 35 °C compost tea.

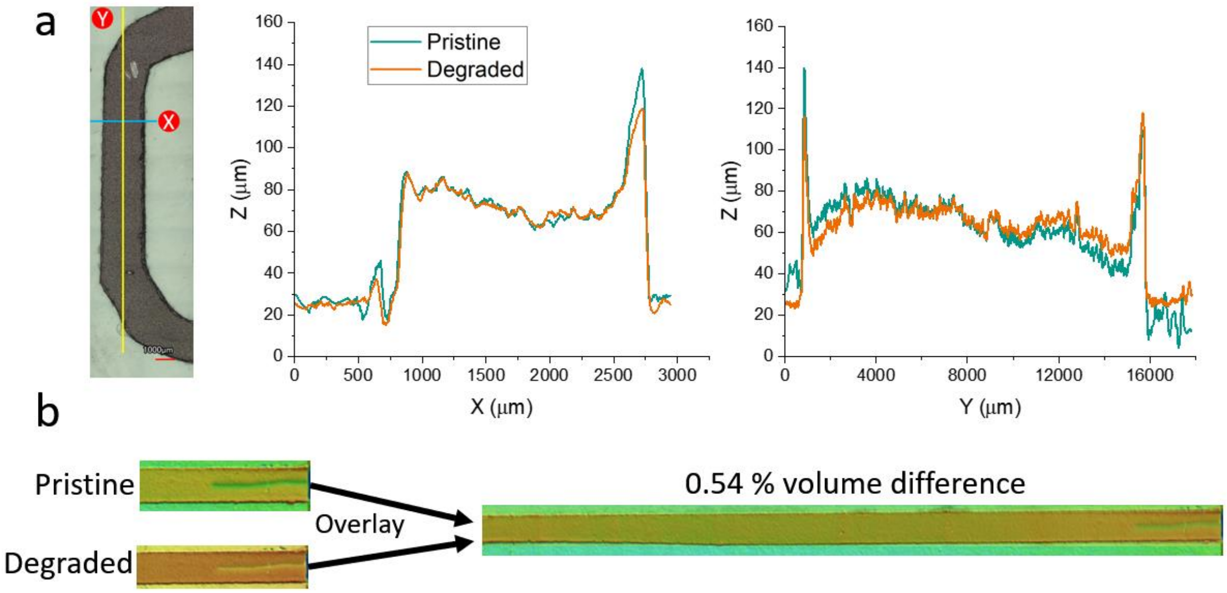

Figure S3: Confocal microscopy of a dry trace before and after degradation shows minimal change to bulk geometry. a) Thickness profiles of lower portion of pristine and degraded PHBV-C trace. b) Overlay of pristine and degraded showed an overall 0.54% change in volume.

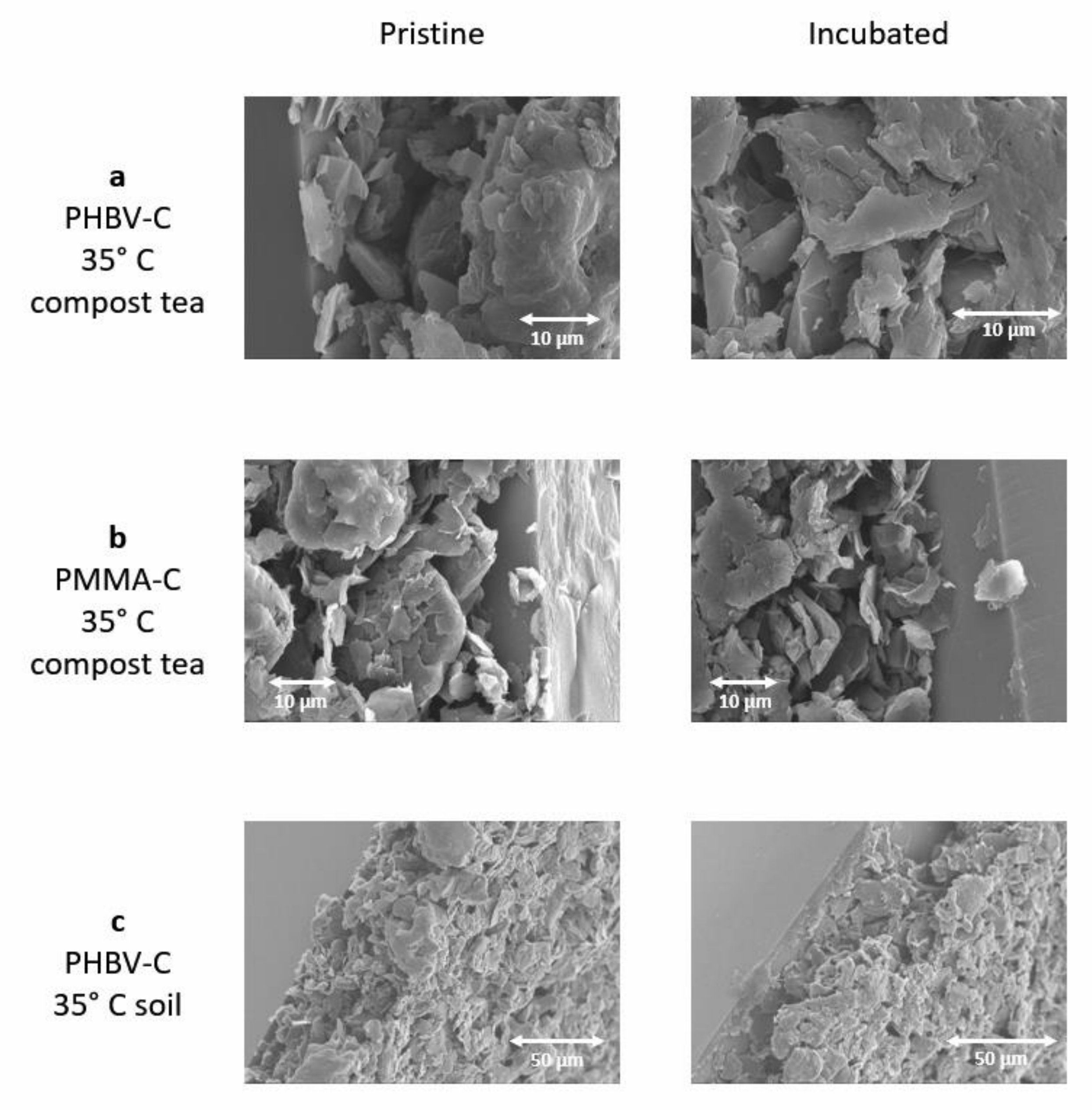

Figure S4: Scanning electron micrographs of pristine traces as compared to traces incubated in different media.

(a) (b)

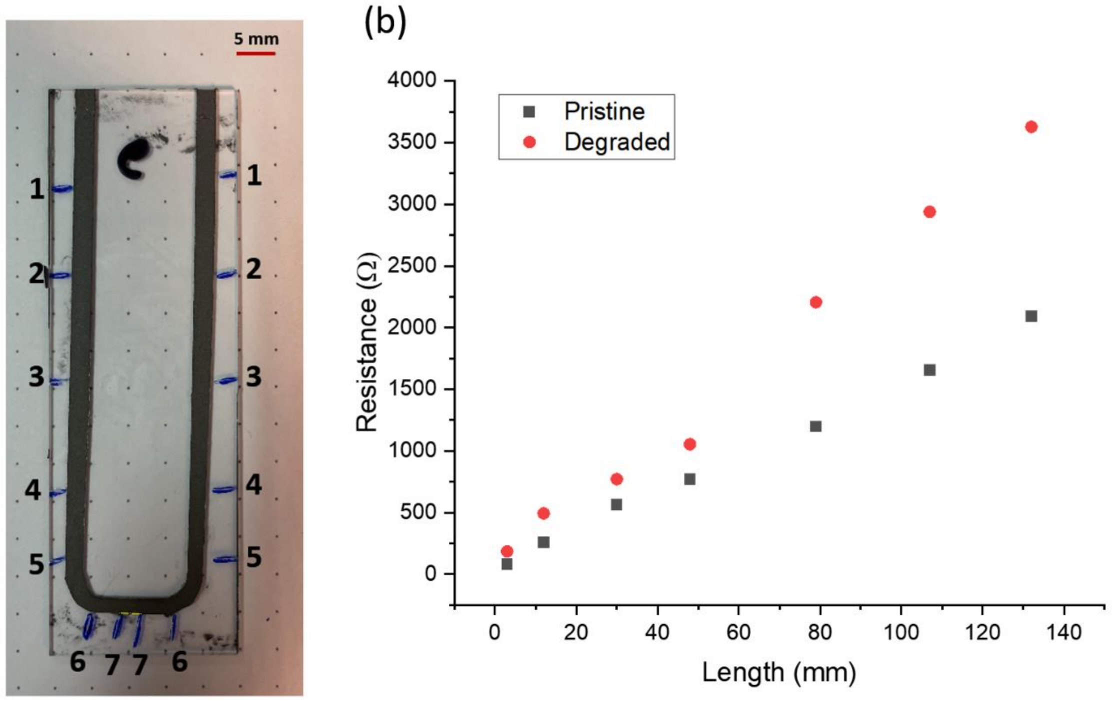

Figure S5: (a) Photograph of PHBV:C trace used for conducting I-V sweeps with electrodes placed at indicated locations. (b) Plot of resistance (derived from linear regression of I-V curve slopes) versus length of trace degraded in 35 °C compost tea for 7 days.

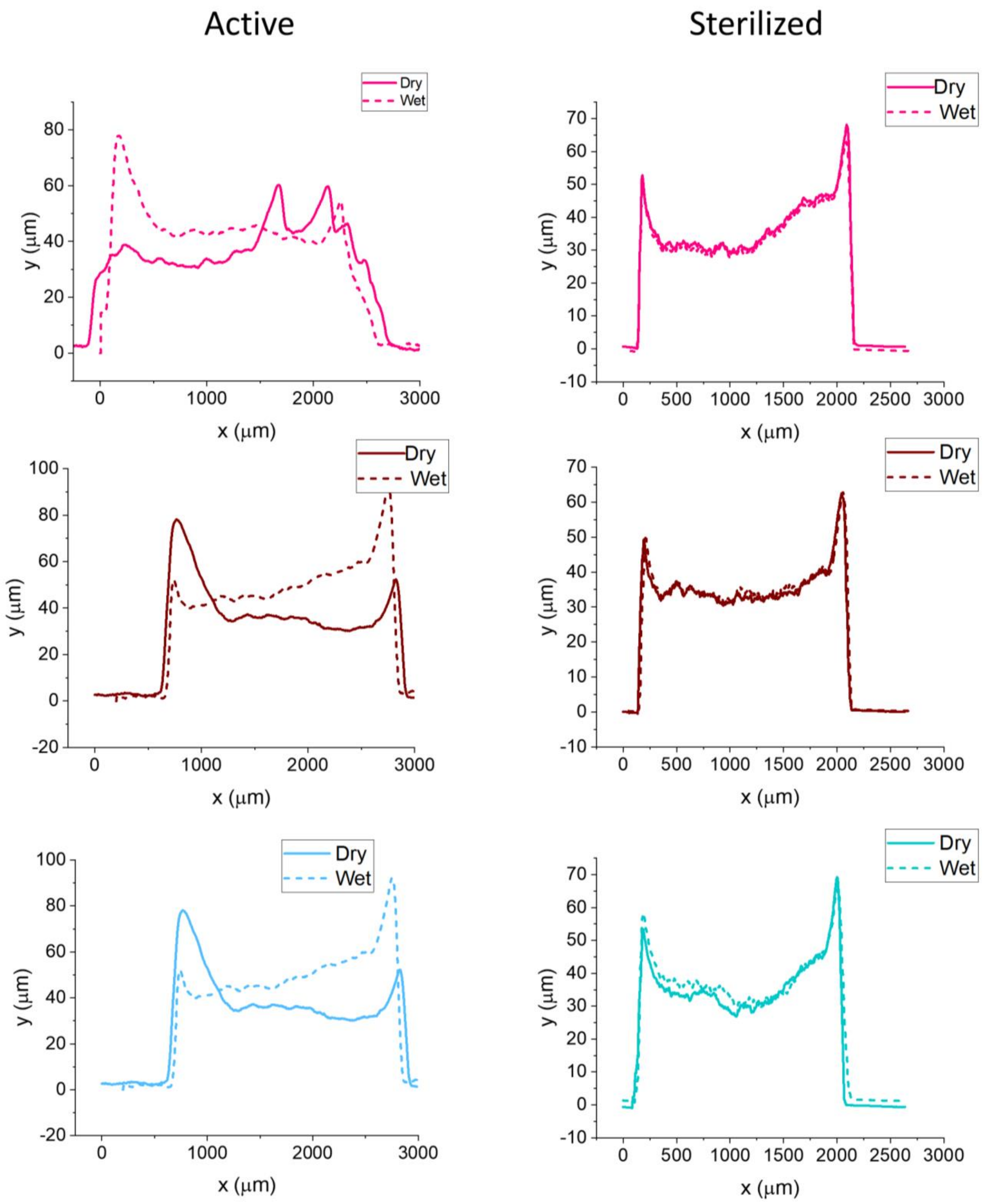

Figure S6: Results of laser confocal profilometry study on trace degraded in microbially active compost tea (left column) and trace incubated in sterilized compost tea (right column). Each line represents an average of 10 measurements taken 50 µm apart at different locations on the respective trace.

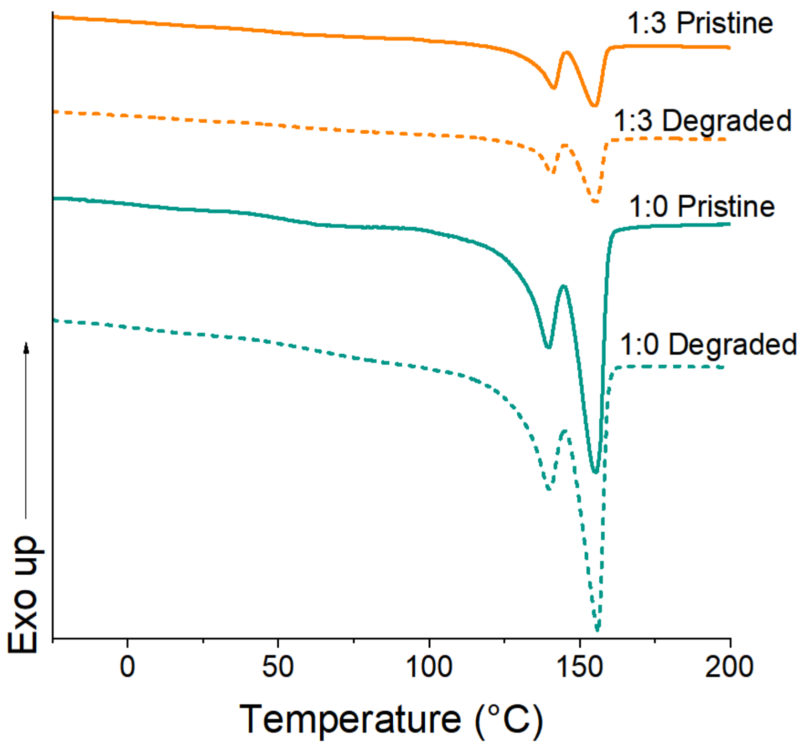

Figure S7: DSC melting curves of pristine and degraded as-purchased PHBV film (1:0) and PHBV:C (1:3) by mass.

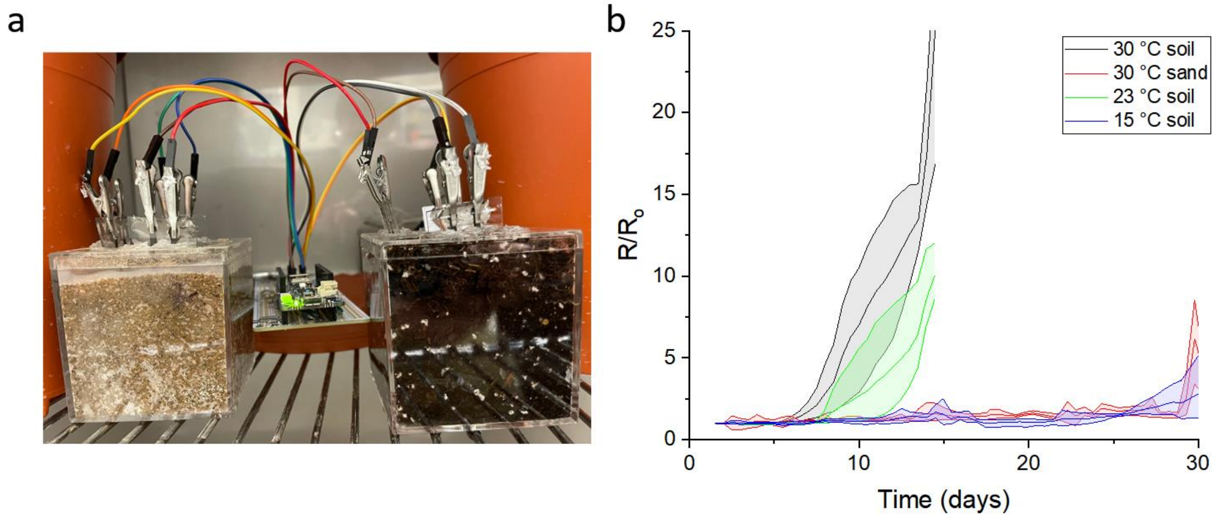

Figure S8: Study to assess the effect of weekly re-watering. a) Experimental setup where conductive traces are submerged in fully saturated sand or potting soil and are connected to the Arduino. b) Plot of normalized resistances of PHBV:C sensors.

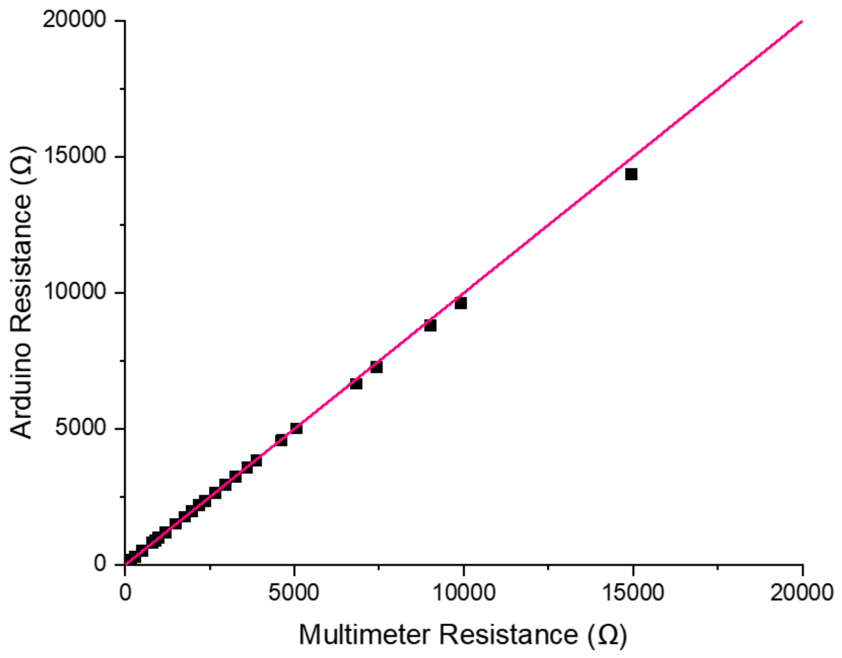

Figure S9: Comparison between Arduino resistance readout versus multimeter resistance readout. Line represents a one-to-one match.
